# Association between carotid artery perivascular fat density and cerebral small vessel disease

**DOI:** 10.18632/aging.203327

**Published:** 2021-07-21

**Authors:** Dan-Hong Zhang, Jiao-Lei Jin, Cheng-Fei Zhu, Qiu-Yue Chen, Xin-Wei He

**Affiliations:** 1 Department of Neurology, Taizhou Central Hospital (Taizhou University Hospital), Taizhou 317700, Zhejiang, China

**Keywords:** perivascular fat density, internal carotid artery, cerebral small vessel disease, lacune, white matter hyperintensity

## Abstract

Studies aiming to identify the significance of the carotid artery perivascular fat density are limited. The present study investigated the distribution pattern of pericarotid fat and its association with imaging markers of cerebral small vessel disease (CSVD). In total, 572 subjects who underwent both neck computed tomography angiography and cranial magnetic resonance imaging were analyzed. The pericarotid fat density near the origin of the internal carotid artery (ICA) and imaging markers of CSVD, such as lacunes, white matter hyperintensities (WMHs) and dilated perivascular spaces (PVSs), were assessed. We found that an increased pericarotid fat density was associated with the presence of lacunes and a higher WMH grade in all subjects, but in the patients with acute ischemic stroke, there was a difference only among the WMH grades. There was no significant difference in the pericarotid fat density in different grades of PVSs. The patients with acute ischemic stroke had a significantly higher mean pericarotid fat density than those without stroke. In conclusion, our study provides evidence suggesting that an increased pericarotid fat density is associated with the presence and degree of WMHs and lacunes. Our findings suggested that features that appear to extend beyond the vessel lumen of the ICA may be linked to CSVD.

## INTRODUCTION

Almost all systemic blood vessels are surrounded by perivascular fat, whose density changes as a surrogate marker of localized inflammation [1].

Perivascular fat encases the carotid artery without an intervening fascial barrier. Limited work has attempted to identify the significance of pericarotid fat. A previous study showed that the fat density around symptomatic internal carotid artery (ICA) stenosis is increased in patients with ischemic events secondary to carotid disease [2]. In addition, a recent study showed that the presence and density of pericarotid fat could be used as an indirect marker of carotid plaque instability [3].

Cerebral small vessel disease (CSVD) is common in the population, especially among elderly individuals [4–6]. Magnetic resonance imaging (MRI) can identify many features of CSVD, including lacunes, white matter hyperintensities (WMHs), and dilated perivascular spaces (PVSs) [4, 7].

The relationship between perivascular fat and adjacent tissues and organs is complex and likely bidirectional. For instance, epicardial adipose tissue is associated with metabolic disorders, arterial wall inflammation and subsequent atherogenesis [7, 8]. In addition, the association with pericardial fat may depend on the particular location of fat relative to the pericardium, and fat distribution surrounding the aorta may also be important [9]. Pathological changes surrounding vessels seem to be associated with nearby parenchymal alterations.

Whether pericarotid fat is correlated with CSVD has not been investigated. The aim of the current study was to investigate the distribution pattern of pericarotid fat and its association with the imaging markers of CSVD.

## RESULTS

### Baseline characteristics

In total, 572 subjects were included. As shown in Table 1, the average age of the patients was 66 (59, 74) years, and 65.2% of the patients were males. The overall mean HU of the pericarotid fat was -77.32±13.03, and the overall maximum HU was -62.48±14.55. Pericarotid fat was weakly correlated with age (mean HU R=0.116 *P*=0.006 and maximum HU R=0.100 *P*=0.017). In addition, the patients with hypertension had a significantly higher mean pericarotid fat density (-76.72±13.13 vs. -79.34±12.55, *P*=0.008, Supplementary Table 1), and the patients with hyperlipidemia had a significantly higher mean pericarotid fat density (-76.40±12.50 vs. -78.75±13.71, *P*=0.040, Supplementary Table 1).

**Table 1:** Baseline characteristics of the participants.

**Characteristics**	**All (n=572)**	**Without AIS (n=195)**	**With AIS (n=377)**	***P***
Age, years	66 (59, 74)	64 (58, 70)	68 (60, 76)	<0.001
Male, n (%)	373 (65.2%)	131 (67.2%)	242 (64.2%)	0.555
Hypertension	440 (76.9%)	141 (72.3%)	299 (79.3%)	0.041
Diabetes mellitus	225 (39.3%)	70 (35.9%)	155 (41.1%)	0.201
Hyperlipidemia	348 (60.8%)	115 (58.7%)	233 (61.8%)	0.444
Coronary heart disease	152 (26.6%)	53 (27.2%)	99 (26.3%)	0.855
Atrial fibrillation	145 (25.3%)	41 (21.0%)	114 (30.2%)	0.018
Current smoking	186 (32.5%)	48 (24.6%)	138 (36.6%)	0.003
Drinking	98 (17.1%)	36 (18.5%)	62 (16.5%)	0.572
Medication				
Antihypertensive therapy	285 (49.8%)	88 (45.1%)	197 (52.3%)	0.089
Antidiabetic therapy	149 (26.0%)	47 (24.1%)	102 (27.1%)	0.497
Statins	125 (21.9%)	40 (20.5%)	85 (22.5%)	0.546
Antithrombotic therapy	145 (25.3%)	38 (19.5%)	107 (28.4%)	0.018
Mean HU of pericarotid fat	-77.32±13.03	-79.64±12.83	-76.11±12.99	0.002
Maximum HU of pericarotid fat	-62.48±14.55	-64.60±14.98	-61.38±14.22	0.013

The values are presented as the mean ± SD or median (interquartile range) for continuous variables and as a number (percentages) for categorical variables.

Abbreviations: AIS, Acute ischemic stroke; HU, Hounsfield Units.

### Pericarotid fat and MRI markers of CSVD

**Figure 1:**
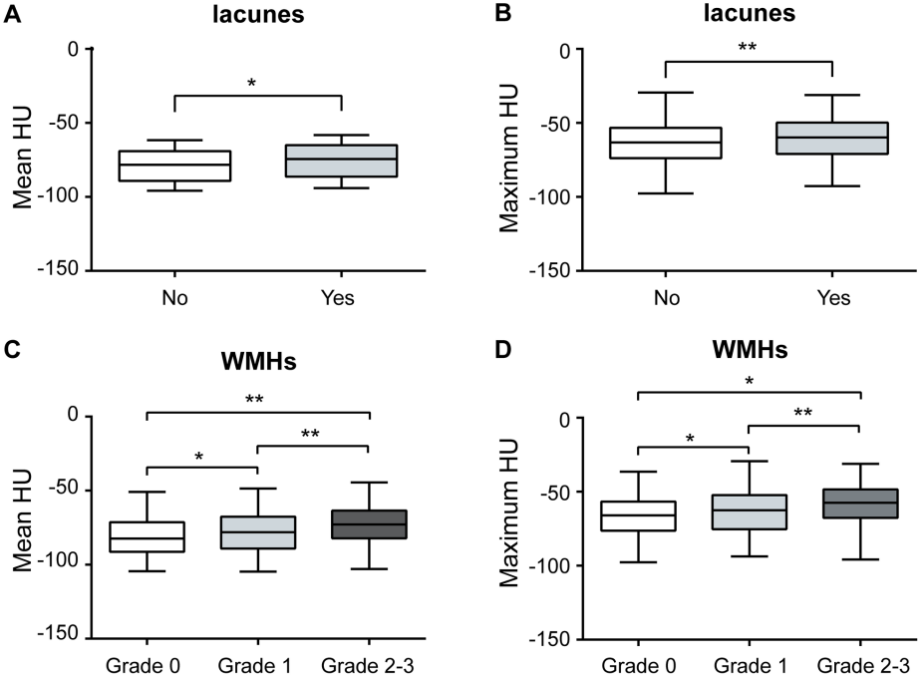
**Association between pericarotid fat and MRI markers of CSVD.** Pericarotid fat density was associated with the presence of lacunes, (**A**) for mean HU, and (**B**) for maximum HU. Pericarotid fat density was associated with the different grades of WMHs, (**C**) for mean HU, and (**D**) for maximum HU. In the box-and-whisker plots, the lower and upper ends of the box represent the 25th and 75th percentiles, and the peripheral lines extending to the outer fences represent the 10th and 90th percentiles, respectively. *<0.05; **<0.01. Abbreviation, WMHs, white matter hyperintensities, HU, hounsfield unit.

An increased pericarotid fat density was associated with the presence of lacunes (mean HU *P*=0.005 and maximum HU *P*=0.023, Tables 2, 3 and Figure 1A, 1B) and a higher WMH grade (both mean and maximum HU *P*<0.001, Tables 2, 3 and Figure 1C, 1D), and both remained significantly different after adjusting for the other clinical parameters, except for the maximum HU in WMHs (Grade 1). Although there was a difference in the maximum HU across different grades of PVSs, the difference disappeared after adjusting for the other clinical parameters (Table 3). There was no significant difference in the mean HU across the different grades of PVSs (Table 2).

**Table 2:** Association between the mean HU of perivascular fat density and neuroimaging markers of CSVD.

**Characteristics**		**N**	**Mean HU**				
			**Value**	***P***		**AOR (95% CI)**	***P***
Lacunes	-	363	-78.35 (-89.13, -69.08)^*^	0.005			
	+	209	-74.39 (-86.33, -65.07)^*^			1.218 (1.038, 1.430)	0.016
WMHs	Grade 0	143	-82.36 (-91.33, -71.25)^*#^	<0.001			
	Grade 1	236	-78.02 (-89.13, -67.58)^*@^			1.366 (1.083, 1.725)	0.009
	Grade 2-3	193	-72.79 (-82.18, -63.45)^#@^			1.396 (1.146, 1.702)	0.001
PVS	Grade 0	141	-78.83±12.35	0.067			
	Grade 1	323	-77.50±13.16			1.066 (0.870, 1.307)	0.538
	Grade 2-4	108	-74.79±13.24			1.189 (0.957, 1.476)	0.118

The results are adjusted for age, sex, hypertension, diabetes, dyslipidaemia, atrial fibrillation, coronary artery disease, smoking, and drinking. Quantitative data were divided into several layers by per standard deviation increase.

For WMHs, ^*^Grade 0 vs Grade 1, <0.05; ^#^Grade 0 vs Grade 2-3, <0.01; ^@^Grade 1 vs Grade 2-3, <0.01.

Abbreviations: CSVD, cerebral small vessel disease; PVS, perivascular spaces; WMHs, white matter hyperintensities; HU, Hounsfield Units; AOR, adjusted odds ratio; CI, confidence interval.

**Table 3:** Association between the maximum HU of perivascular fat density and neuroimaging markers of CSVD.

**Characteristics**		**N**	**Maximum HU**				
			**Value**	***P***		**AOR (95% CI)**	***P***
Lacunes	-	363	-63.53±14.73^#^	0.023			
	+	209	-60.66±14.10^#^			1.173 (1.020, 1.386)	0.047
WMHs	Grade 0	143	-66.44±13.72^*^	<0.001			
	Grade 1	236	-63.43±15.10^#^			1.126 (0.902, 1.405)	0.294
	Grade 2-3	193	-58.38±13.47^*#^			1.356 (1.118, 1.645)	0.002
PVS	Grade 0	141	-64.10±13.54^*^	0.025			
	Grade 1	323	-62.86±14.85			1.033 (0.845, 1.263)	0.753
	Grade 2-4	108	-59.22±14.55^*^			1.257 (1.012, 1.561)	0.083

The results are adjusted for age, sex, hypertension, diabetes, dyslipidaemia, atrial fibrillation, coronary artery disease, smoking, drinking, and drugs. Quantitative data were divided into several layers by per standard deviation increase.

For Lacunes, - vs +, ^#^<0.01.

For WMHs, ^*^Grade 0 vs Grade 2-3, <0.05; ^#^Grade 1 vs Grade 2-3, <0.01.

For PVS, ^*^Grade 0 vs Grade 2-4, <0.05.

Abbreviations: CSVD, cerebral small vessel disease; PVS, perivascular spaces; WMHs, white matter hyperintensities; HU, Hounsfield Units; AOR, adjusted odds ratio; CI, confidence interval.

There were 377 (65.9%) patients with acute ischemic stroke (AIS). The patients with AIS had a significantly higher pericarotid fat density than those without AIS (mean HU *P*=0.002 and maximum HU *P*=0.013, Table 1). The difference in the mean HU remained after adjusting for the different clinical parameters between the groups, while the difference in the maximum HU disappeared (mean HU AOR (95% CI), 1.179 (1.003-1.387), *P*=0.040 and maximum HU *P*=0.097).

In the sensitivity analysis of the 192 patients without stroke, there was a significantly higher pericarotid fat density in the lacune group and the higher WMH grade group (Table 4). In 377 patients with stroke, the pericarotid fat density was higher in the high WMH grades, while there was no significant difference by lacunes and PVSs (Table 4).

**Table 4:** Association between the perivascular fat density and neuroimaging markers of CSVD in patients with or without stroke.

**Characteristics**		**Without AIS**			**With AIS**	
		**Mean HU**	**Maximum HU**		**Mean HU**	**Maximum HU**
Lacunes	-	-81.50±12.22^#^	-66.44±14.68^#^		-76.70±13.15	-61.77±14.51
	+	-75.31±13.29^#^	-75.31±14.91^#^		-75.23±12.73	-60.79±13.81
WMHs	Grade 0	-83.19±12.05^#^	-69.14±13.55^#^		-80.81±11.69^*#^	-65.16±13.68^*^
	Grade 1	-80.62±12.69^*^	-65.36±15.34^*^		-76.04±13.27^*^	-62.00±14.83
	Grade 2-3	-74.29±12.42^#*^	-58.79±13.92^#*^		-73.02±12.69^#^	-58.24±13.37^*^
PVS	Grade 0	-80.63±12.00	-66.32±13.37		-77.65±12.50	-62.64±13.54
	Grade 1	-80.55±13.18	-65.63±15.59		-76.08±12.95	-61.57±14.35
	Grade 2-4	-75.58±12.62	-59.14±14.69		-74.38±13.63	-59.27±14.59

For Lacunes, ^#^<0.01. For WMHs, ^*^<0.05; ^#^<0.01.

Abbreviations: AIS, Acute ischemic stroke; CSVD, cerebral small vessel disease; PVS, perivascular spaces; WMHs, white matter hyperintensities; HU, Hounsfield Units.

### Pericarotid fat density in patients with extracranial ICA stenosis

**Figure 2:**
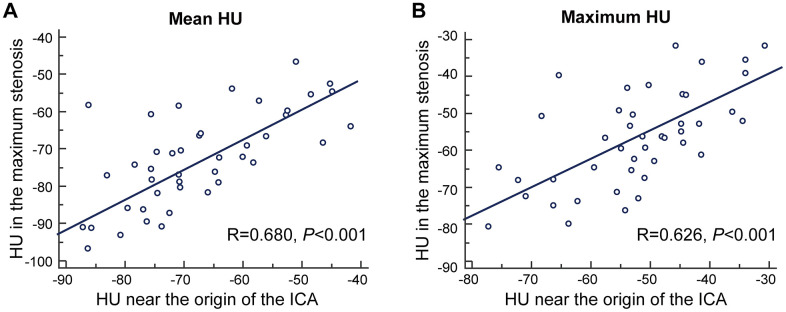
**The correlation between density of pericarotid fat in the maximum stenosis slice and near the origin of the ICA.** (**A**) for mean HU. (**B**) for maximum HU. Abbreviation, HU, hounsfield unit, ICA, internal carotid artery.

Forty-three subjects had extracranial ICA stenosis, and we recorded the value at the maximum stenosis slice. The pericarotid fat in the slice with maximum stenosis had a higher density than that near the origin of the ICA (mean HU -67.33±12.34 vs. -72.62±12.58, *P*=0.008 and maximum HU -52.39±11.48 vs. -56.89±13.05, *P*=0.008). In addition, the mean HU and maximum HU in these two places were strongly correlated (R=0.680, *P*<0.001 and R=0.626, *P*<0.001, Figure 2).

Because the number of patients with extracranial ICA stenosis was small, we could not perform a separate analysis of the association between the perivascular fat density and MRI markers of CSVD limited to these patients.

## DISCUSSION

This study provides an objective assessment of the association between the pericarotid fat density and imaging markers of CSVD. An increased pericarotid fat density was associated with the presence of lacunes and a higher WMH grade in all subjects. There was no significant difference in the pericarotid fat density across different grades of PVSs. The patients with AIS had a significantly higher pericarotid fat density. The pericarotid fat density in the maximum stenosis slice was higher than that near the origin of the ICA in patients with extracranial ICA stenosis.

Although some previous studies have suggested that visceral fat accumulation is related to the presence of various markers of CSVD, including WMHs and lacunes [10–12], it is more likely to be mediated by adiposity-related risk factors, such as hypertension, diabetes or dyslipidemia. It is generally known that visceral adipose tissue accumulation is positively associated with cardiovascular disease. In the present study, although we found that the pericarotid fat density was associated with CSVD risk factors, such as age, hypertension and hyperlipidemia, the association between the pericarotid fat density and CSVD remained significant after adjusting for these clinical parameters. Thus, pericarotid fat is an independent risk factor for CSVD.

We found that the pericarotid fat density was associated with the presence of lacunes and higher WMH grades. Although the relationship between carotid artery disease and CSVD has been gradually revealed [13–15], the pathophysiologic mechanisms underlying the relationship between pericarotid fat and CSVD have not been fully established. Increasing studies have identified that perivascular fat has endocrine and paracrine functions, and the pathophysiological characteristics seem to be distinct in different anatomical locations and metabolic statuses [7, 8, 16].

One possible explanation is that pericarotid fat is a vascular risk factor that predisposes individuals to developing CSVD. Previous studies have revealed that both asymptomatic and symptomatic carotid atherosclerosis and compliance are associated with imaging markers of CSVD [7, 17, 18], and this phenomenon is more obvious in the ipsilateral hemisphere with ICA stenosis than the contralateral hemisphere [18]. It is conventionally believed that perivascular fat has a detrimental effect on the vessel [8, 19]. For example, inflammatory changes in the fat surrounding the coronary artery are associated with high-risk plaque changes and coronary artery disease [2, 20]. In addition, an increased density of pericarotid fat was recently described in high-risk vulnerable carotid atherosclerotic plaque [2]. Thus, it can be inferred that the structural and functional changes in upstream large arteries affected by perivascular fat may alter the hemodynamics of small vessels downstream in the brain and might be linked to the pathogenesis of CSVD [21]. Notably, the relationship between the vascular wall and the surrounding adipose tissue is complex and likely bidirectional; for example, inflamed human vessels exert paracrine effects on the surrounding perivascular fat, preventing local intracellular lipid accumulation [22].

Another possible explanation is the direct communication between adipose tissue and the surrounding tissue. The perivascular fat of the ICA may have a profound influence on adjacent tissues through locally secreted biochemical factors, such as a paracrine fat organ in other parts [8, 9, 19]. Recent data support the role of perivascular fat density changes as a histopathologic marker of low-grade inflammation [23, 24]. Inflammation could impair endothelial function, leading to damaged regulation of vascular tone and the vasodilatory response, resulting in insufficient perfusion in the small perforating artery area and causing parenchymal changes, which are two potential mechanisms accounting for WMHs and lacunes.

We did not observe a significant correlation between pericarotid fat and the degree of PVSs. PVSs are extensions of the extracerebral fluid-filled spaces that follow the typical course of a vessel as it passes through gray or white matter, which is considered a passive anatomical structure secondary to transient vasoconstriction and vasodilation of the vessel it surrounds [25]. Our study suggested that pericarotid fat may not play a major role in the formation of dilated PVSs.

We found that the patients with AIS had a significantly higher mean pericarotid fat density than those without AIS. Our finding is similar to recent findings suggesting an increased density in the pericarotid fat surrounding the stenotic ICAs of patients with ischemic events compared with that of patients who were asymptomatic [18]. As mentioned above, pericarotid fat is directly wrapped around the carotid artery, and a higher density of pericarotid fat is a marker of carotid atherosclerotic plaque instability and vascular inflammation, which are risk factors for ischemic stroke. Recent studies have suggested that certain vulnerable plaques may present a risk factor for acute embolic infarction even though they do not cause any hemodynamically significant stenosis [26]. Our findings may have implications for identifying vulnerable plaques. Previous studies demonstrated that inflammatory signals secreted by the vascular wall of vulnerable plaques may prevent lipid accumulation by influencing the biological processes of adipocyte differentiation, proliferation and lipolysis [27, 28]. Thus, the density of perivascular fat could be increased due to decreased lipid accumulation under active vessel inflammation. Similarly, perivascular fat surrounding the proximal segments of the major coronary arteries had a striking value in the prognosis of cardiac death and nonfatal myocardial infarction and was associated with an increased risk of fatal heart attacks [29, 30]. The above results highlight the need for more tailored therapy for a subset of patients with a higher perivascular fat density to prevent recurrent stroke.

In the patients with extracranial ICA stenosis, we found that the pericarotid fat density in the maximum stenosis slice had a larger mean HU and maximum HU than those near the origin of the ICA. A previous study found a significantly increased pericarotid fat density around the stenotic ICA compared with that around the nonstenotic ICA on the same axial slice [18]. Combined with our research, these results further suggest that the presence of significant carotid artery stenosis is associated with increased perivascular fat inflammation. In addition, we found that the pericarotid fat density has correlations between the origin of the ICA and maximum stenosis. Because the origin of the ICA can easily and consistently be located in individuals, the density of fat in this place appears to be a marker of pericarotid fat.

Our study has some limitations. First, due to the cross-sectional nature of the study, causality cannot be determined. Future prospective studies could be helpful in confirming the cause-effect relationship. Second, we included a large proportion of stroke patients and limited the age to between 50 and 80 years. The prevalence of WMHs and lacunar infarcts might be overestimated, and these patients were older. Neck CTA is usually performed because of suspected cerebrovascular disease. In addition, the MR images were evaluated by a single observer, and such analyses could be more rigorous when performed by several observers. Third, because Asian populations have a higher proportion of intracranial atherosclerotic stenosis [31, 32], which is also related to the imaging markers of CSVD, intracranial atherosclerotic stenosis should be considered. Fourth, the measurement of fat density on CT can be affected by variability in the ROI placement. We chose to measure the average from bilateral ICAs from 3 discontinuous sections to reduce the degree of variation.

Nevertheless, to the best of our knowledge, this study was the first to investigate the association between pericarotid fat and CSVD and suggest that features that appear to extend beyond the vessel lumen of the ICA may be linked to CSVD. This information has implications for whether targeted medical intervention for perivascular fat can be effective in preventing diseases of the carotid artery and progression of CSVD.

In conclusion, our study provides evidence suggesting that an increased pericarotid fat density is associated with the presence and degree of WMHs and lacunes. It is necessary to identify potential biological pathways supporting the association between pericarotid fat changes and CSVD.

## MATERIALS AND METHODS

### Patient recruitment

We screened patients consecutively at our department from January 1, 2016, to December 31, 2019, to identify subjects meeting the following inclusion criteria: 1) an interval between the screened neck CTA and cranial MRI no longer than 3 months and 2) an age between 50 and 80 years. The exclusion criteria were as follows: 1) severe malacia lesion, extensive or old infarction, hemorrhage, atrophy or tumor; 2) other diseases that may cause white matter lesions, such as multiple sclerosis, vasculitis, and connective tissue diseases; or 3) poor imaging quality or partially missing images.

This study was approved by the ethics committee of the Taizhou Central Hospital (approval number, 2020L-12-04). The study protocol conforms to the ethical guidelines of the 1975 Declaration of Helsinki. Informed patient consent was exempted because this was a retrospective study based on routine clinical data.

### Data collection and determination

The demographics, clinical features, and vascular risk factors were extracted from the patients’ medical records. The risk factors were defined as follows: hypertension (systolic/diastolic blood pressure > 140/90 mmHg over repeated measurements or a medical history of hypertension), diabetes mellitus (fasting blood glucose > 7.0 mmol/L, hemoglobin A1c > 6.5%, self-reported diabetes mellitus, or the use of oral antidiabetic drugs or insulin), hyperlipidemia (serum triglycerides > 1.7 mmol/L, low-density lipoprotein > 3.4 mmol/L, high-density lipoprotein cholesterol < 0.8 mmol/L, or the use of statins), current smoking (currently smoking or quit smoking within 1 year of admission), and drinking (> 2 standard alcoholic beverages consumed per day).

### Neuroimaging acquisition and processing

Computed tomography angiography (CTA) was performed using a 64-slice Discovery CT750 HD (GE, USA) with the following parameters: 100 kVp, 3 mAs, section thickness 0.625 mm, interval 0.625 mm, and display field of view (DFOV) 250 × 250 mm. Intravenous iodinated contrast (Ioversol Injection, 1.5-2 ml/kg; Hengrui Medicine Co., Ltd., China) was administered at a rate of 4.0 mL/s.

The MRI examinations were performed using a 1.5T MR-Signa HDx MRI system (GE, USA). The MRI images were obtained after axial scanning using the following parameters: time repetition (TR)/time echo (TE)=8200/109 ms, slice thickness (ST) = 5 mm, DFOV = 165 × 240 mm for T2-weighted images; TR/TE = 464/14 ms, ST = 4 mm, and DFOV = 225 × 240 mm for T1-weighted images; and TR/TE = 3400/94 ms, ST = 5 mm, and DFOV = 230 × 230 mm for diffusion-weighted imaging (DWI).

### Pericarotid fat density analysis

**Figure 3:**
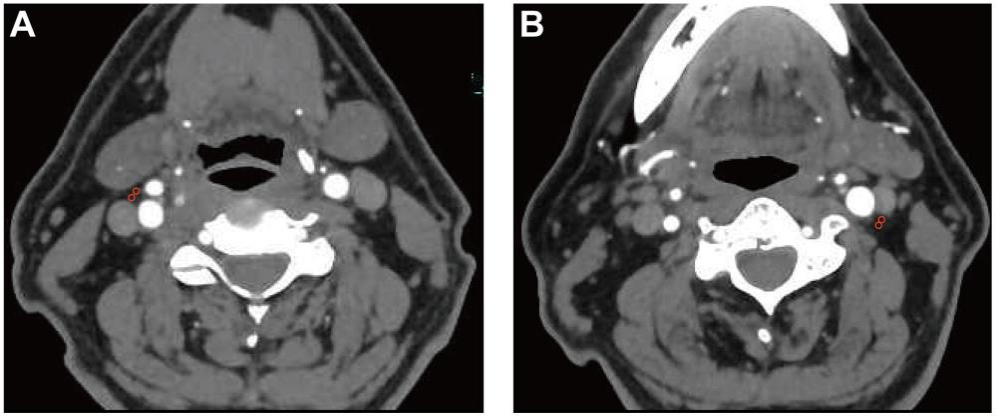
**Pericarotid fat density analysis.** Two regions of interest (3 mm^2^ in diameter) were placed in the pericarotid fat surrounding the origin of the internal carotid artery. (**A**) right internal carotid artery. (**B**) left internal carotid artery.

The density of the pericarotid fat surrounding the extracranial ICA can be measured via Hounsfield units (HU) on routine CTA imaging. We referred to an established approach previously described in [2] using predefined image display settings (window width, 500 HU; window center, 100 HU). We placed 2 regions of interest (ROIs) (3 mm^2^ in diameter) in the perivascular fat on both sides of the ICAs (Figure 3). The ROIs were placed at least 1 mm from the outer margin of the carotid artery wall to exclude the carotid artery wall and surrounding soft-tissue structures. The HU values were recorded from 3 discontinuous slices near the origin of the ICA, and then, the mean and maximum HU values were measured. In addition, if North American Symptomatic Carotid Endarterectomy Trial (NASCET)-defined ICA stenosis was present, we recorded the value at the maximum stenosis slices [33].

The evaluation of the density of the pericarotid fat was performed by a neuroradiologist blinded to the clinical data. To test the reliability of the measurements, a second neuroradiologist blinded to the clinical data and initial measurements re-evaluated a subset of patients with extracranial ICA stenosis. The average value of the two data points was calculated and used. The intraclass correlation coefficient (ICC) values of the mean and maximum HU from the two observers were 0.808 (0.67, 0.89) and 0.842 (0.73, 0.91), respectively. A Bland-Altman analysis was performed as shown in Supplementary Figure 1. Our measurements were simple and showed high reproducibility, suggesting that interreader differences are likely relatively modest using this technique.

### Assessment of MRI markers of CSVD

All imaging markers of CSVD were defined according to the neuroimaging standards as usual [7, 34]. The presence of lacunes, WMHs, and PVSs was observed independently outside the acute infarct area (based on DWI). Briefly, lacunes were defined as round or ovoid, subcortical, fluid-filled cavities (signal similar to cerebrospinal fluid) of 3-15 mm in diameter situated in the subcortical white matter, basal ganglia, or brain stem. Cerebral WMH was defined as a focal ≥3 mm lesion without definite hypointensity on T1-weighted images and with hyperintensity on fluid-attenuated inversion recovery (FLAIR) and T2-weighted images and rated according to the Fazekas scale from 0 to 3 [35]. PVSs were defined as cerebrospinal fluid (CSF)-like signal lesions that were round, ovoid, or linear and 1-3 mm in diameter situated in the centrum semiovale and basal ganglia and rated on a validated semiquantitative scale from 0 to 4 [36]. In this study, we counted PVSs only in the basal ganglia because the PVSs in this region seemed to be specifically associated with CSVD [37]. One neuroradiology board-certified expert in our hospital who was blinded to the clinical data reviewed all brain MR images.

### Statistical analysis

The normally distributed data are presented as the mean ± standard deviation (SD), and the nonnormally distributed data are presented as the median (interquartile range). The categorical data are presented as frequencies and percentages. The clinical and neuroimaging characteristics of the groups were compared using Student’s *t*-test, paired *t*-test, analysis of variance with Bonferroni correction, Mann–Whitney *U* test or Pearson's chi-square test as appropriate. The correlations were measured using a Spearman correlation analysis. We calculated the ICC and corresponding 95% CI to evaluate the degree of interreader reliability. In the multivariate analyses, the associations between the perivascular fat density (independent variable) and MRI markers of CSVD (dependent variables) were investigated using a binary logistic regression (for the presence of lacunes) and ordinal logistic regression (for WMHs and PVSs). The severity of WMHs and PVSs was trichotomized because of the small number of samples with a more severe degree (mild [degree 0], moderate [degree 1], and severe [degrees 2-3 for WMHs and degrees 2-4 for PVSs]). The quantitative data were divided into several layers per standard deviation increase. The results are expressed as the adjusted ORs (multivariate analysis) along with their 95% confidence intervals (CIs). All data were analyzed using SPSS 20.0 (IBM, Chicago, IL, USA). Two-sided *P*-values<0.05 were considered statistically significant if not otherwise specified.

## Supplementary Material

Supplementary Figure 1

Supplementary Table 1
