# Supplementary material for: Association between carotid artery perivascular fat density and cerebral small vessel disease
Source: Aging (Albany NY). 2021 Jul 21;13(14):18839–51. doi: 10.18632/aging.203327 (PMC8351687; doi:10.18632/aging.203327)
Supplement: Supplementary Figure 1 [file aging-13-203327-s001.pdf]

SUPPLEMENTARY FIGURE

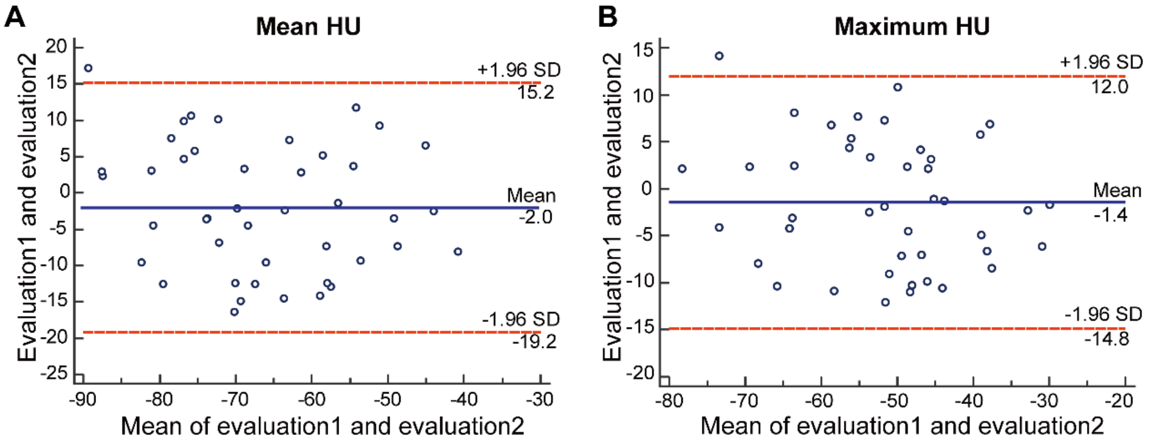

Supplementary Figure 1. The Bland-Altman analysis for the two evaluation. (**A**) for mean HU, and (**B**) for maximum HU. Abbreviation, HU, hounsfield unit.
