# Supplementary material for: Association between carotid artery perivascular fat density and cerebral small vessel disease
Source: Aging (Albany NY). 2021 Jul 21;13(14):18839–51. doi: 10.18632/aging.203327 (PMC8351687; doi:10.18632/aging.203327)
Supplement: Supplementary Table 1 [file aging-13-203327-s002.pdf]

SUPPLEMENTARY TABLE

Supplementary Table 1. Association between the perivascular fat density and CSVD risk factors

Characteristics	Mean HU			Maximum HU		
	No	Yes	*P*	No	Yes	*P*
Male, n (%)	-76.89±13.34	-77.55±12.88	0.564	-61.52±14.68	-62.99±14.48	0.249
Hypertension	-79.34±12.55	-76.72±13.13	**0.043**	-64.63±13.96	-61.84±14.68	0.054
Diabetes mellitus	-78.01±12.73	-76.26±13.45	0.117	-62.99±14.57	-61.70±14.53	0.301
Hyperlipidemia	-78.75±13.71	-76.40±12.50	**0.040**	-63.82±15.47	-61.62±13.89	0.079
Coronary heart disease	-77.58±13.00	-76.59±13.13	0.423	-62.77±14.60	-61.68±14.46	0.426
Atrial fibrillation	-77.76±12.82	-76.14±13.57	0.188	-63.04±14.40	-60.99±14.91	0.134
Current smoking	-77.87±13.22	-76.18±12.60	0.148	-63.05±14.80	-61.30±13.99	0.179
Drinking	-77.34±13.05	-77.23±13.03	0.941	-62.36±14.70	-63.06±13.88	0.668
